# Supplementary material for: The net effect of wealth on health—Investigating noncommunicable diseases mortality in the context of regional affluence
Source: PLoS One. 2023 Nov 6;18(11):e0293887. doi: 10.1371/journal.pone.0293887 (PMC10627448; doi:10.1371/journal.pone.0293887)
Supplement: Attachment — Submitted filename: Response to Reviewers.docx [file pone.0293887.s002.docx]

Dear Editor,

Thank you for giving us the opportunity to revise the paper. Appreciating the valuable comments of the referees’, we hope that we were able to improve our manuscript and address all the concerns thoroughly. We genuinely believe this helped us to improve our paper. We hope that the manuscript is now suitable for publication in Plos One.

We would like to go through the reviewers’ and editorial comments regarding the manuscript briefly.

**________________________________________**

**5. Review Comments to the Author**

**Reviewer #1:**

**- Intro: “This advantage predominantly stems from better education of the general population, higher quality of healthcare specialists, more advanced medical technology financed through general taxes and common social or private medical insurance”. This statement needs to be backed by evidence/reference.**

- *This statement has been backed by the references [10], [19], [20].*

**- Intro: “Overall, a higher level of wealth affects the individual and the population's health in two opposite ways”. Again, more evidence/ref is required here on how these assumptions/suggestions are made.**

- *This statement has been backed by the references [15], [16], [21].*

**- Results and discussions: the findings are well presented. One area of development could be to enrich the discussion further with contextualising external determinants of health disparities and providing some causal explanations. For example, the authors state that some types of neoplasms have lower or decreasing mortality rates in countries with higher Human Development Index, however further analysis of this connection would add further insight into the methodology and results.**

- *The discussion has been elaborated and additional context has been provided and backed by the references [41], [42], [43], [44], [452].*

**- Conclusion: the whole section lack any comparison with similar literature to situate the paper’s finding in the literature. Some of the papers that came to mind are:**

**https://www.sciencedirect.com/science/article/pii/S0277953613000312?casa_token=L6R5_4yTgmwAAAAA:e5fAKRX8e11vbm1eI98HL4FHutzLfOCfIh_XL-F6XxIt32GOCICPzs-LXy6zhTkqTnLS-FwIZw**

**https://www.thelancet.com/journals/lancet/article/PIIS0140-6736(09)60098-2/fulltext**

[**https://www.sciencedirect.com/science/article/pii/S0167629603000596?casa_token=dFiUW-aL8SIAAAAA:x0qGXKlf5mci44lkQGHTb3Lp5jPQsSNTrNyG6655J7m96HIc9vbzjtFpiJuI5xEaXdv98a4NaQ**](https://www.sciencedirect.com/science/article/pii/S0167629603000596?casa_token=dFiUW-aL8SIAAAAA:x0qGXKlf5mci44lkQGHTb3Lp5jPQsSNTrNyG6655J7m96HIc9vbzjtFpiJuI5xEaXdv98a4NaQ)

- *A comparison with similar literature has been done - [19], [45], [60], [61], [62], [63], [64], [65].*

**Generally the paper seem to make good use of operational reports (WHO reports specially), but is limited when it comes to literature review. More lit review will enrich the paper.**

- *The literature review has been added in all sections.*

**- Finally, the language and style should be adjusted to ensure it meets the standards for academic British English.**

- *Professional proofreading has been performed on the revised version of the manuscript by The Cambridge Proofreading & Editing LLC.*

**Reviewer #2:**

***The title should include mortality, not health.**

*- The title has been changed and includes ‘mortality’, i.e. “The Net Effect of Wealth on Health—Investigating Noncommunicable Diseases Mortality in the Context of Regional Affluence”.*

*** There are no policy implications in the abstract.**

*- The policy implications have been added in the abstract.*

*** Lines (51-59) didn't include any references.**

*- Additional information with appropriate references has been added: [3], [7], [9], [11], [17], [18].*

*** In text citations need revision according to the journal requirements.**

*- The citations have been corrected according to the journal requirements.*

***The paragraph begins with line 201 needs more explanation for the method.**

- *The method has been explained in detail:*

*“Linear functions* $f_{1}$ *and* $f_{2}$ *obtained by the joinpoint regression method:*

${MR}_{i}^{d}\left( GDP_{i};\alpha_{0},\alpha_{1},\alpha_{2}, {GDP}^{*} \right)=\left\{ \begin{matrix} f_{1}\left( GDP_{i} \right)+\epsilon_{i}, for i\in R{}_{1} \\ f_{2}\left( GDP_{i} \right)+\epsilon_{i}, for i\in R{}_{2} \end{matrix} \right.$

*can be extrapolated to the regions with GDP outside their respective domains: R_1_ and R_2_. That is, the theoretical extension of the function* $f_{2}$ *covers now regions with lower GDP (R_1_), where theoretical extension of the function* $f_{1}$ *is applied to the regions with higher GDP (R_2_). Operating on those theoretical functions' extrapolations and the empirical values, by calculating the difference between them, we can acquire two net effect values: the net effect of poverty (𝑁𝐸_𝑃) and of affluence (*$NE\_A$*).”*

***Please add** **asterisks in table 3 to show the significance of coefficients.**

*- The* *significance asterisks in Tab. 3, as well as Tab. 2 have been added.*

*** in line 341, please change "determinates" to "determinants".**

*- Done.*

*** You need to add titles of x and y axis in Figure 4.**

*- The titles of x and y axis has been added in Fig. 4.*

*** Please mention the assumptions of jointpoint regression, and if it was tested before the implementation of the model.**

- Appropriate assumptions have been tested, and a proper statement has been added:

**“***We assume that the error terms (*$\epsilon_{i}$*) are randomly distributed, independent with zero mean and a common bound for variances* $\sigma^{2}$ *for both subsets.* *This model can be estimated with an adjusted ordinary least squares method, by following the general theory of Pötscher and Prucha [39]. The resulting M-estimator turns out to be consistent under the same assumptions as standard OLS for the regressors and with the additional requirement of the corresponding parameter space being bounded***.”**

*** In line 92, add the reference of EUROstat database.**

*- The reference of Eurostat database has been added – [22]*

-------------

**Journal Requirements:**

**When submitting your revision, we need you to address these additional requirements.**

**1. Please ensure that your manuscript meets PLOS ONE's style requirements, including those for file naming.**

*- Corrected according to PLOS ONE's style requirements.*

**2. We note that you have stated that you will provide repository information for your data at acceptance. Should your manuscript be accepted for publication, we will hold it until you provide the relevant accession numbers or DOIs necessary to access your data. If you wish to make changes to your Data Availability statement, please describe these changes in your cover letter and we will update your Data Availability statement to reflect the information you provide.**

*- The data file has been provided.*

**3. We note that Figures 1-3 and 5 in your submission contain [map/satellite] images which may be copyrighted. All PLOS content is published under the Creative Commons Attribution License (CC BY 4.0), which means that the manuscript, images, and Supporting Information files will be freely available online, and any third party is permitted to access, download, copy, distribute, and use these materials in any way, even commercially, with proper attribution.**

*- All the maps in the manuscript are of our own compilation using Eurostat shapefiles of administrative borders (under © EuroGeographics for the administrative boundaries). Fig. 1-3 have been created in GeoDa software (under GNU Affero General Public License version 3) and Fig. 5 in ArcMap (under Education Institutional Small International Agreement version 11.1) The software and shapefiles are granted on the condition that the data will not be used for commercial purposes and that the source will be acknowledged – provided in the figures’ description.*

**4. Please review your reference list to ensure that it is complete and correct.**

- *We have reviewed the reference list to ensure it is complete and correct.*
